# Cardiac Channelopathies and Sudden Death: Recent Clinical and Genetic Advances

**DOI:** 10.3390/biology6010007

**Published:** 2017-01-29

**Authors:** Anna Fernández-Falgueras, Georgia Sarquella-Brugada, Josep Brugada, Ramon Brugada, Oscar Campuzano

**Affiliations:** 1 Cardiovascular Genetics Center, IDIBGI, Girona 17190, Spain; afernandez@gencardio.com (A.F.-F.); ramon@brugada.org (R.B.); 2 Arrhythmias Unit, Hospital Sant Joan de Déu, University of Barcelona, Barcelona 08950, Spain; georgia@brugada.org (G.S.-B.); jbrugada@clinic.ub.es (J.B.); 3 Medical Sciences Department, School of Medicine, University of Girona, Girona 17071, Spain; 4 Familial Cardiomyopathies Unit, Hospital Josep Trueta, Girona 17007, Spain

**Keywords:** sudden cardiac death, arrhythmias, channelopathies, genetics

## Abstract

Sudden cardiac death poses a unique challenge to clinicians because it may be the only symptom of an inherited heart condition. Indeed, inherited heart diseases can cause sudden cardiac death in older and younger individuals. Two groups of familial diseases are responsible for sudden cardiac death: cardiomyopathies (mainly hypertrophic cardiomyopathy, dilated cardiomyopathy, and arrhythmogenic cardiomyopathy) and channelopathies (mainly long QT syndrome, Brugada syndrome, short QT syndrome, and catecholaminergic polymorphic ventricular tachycardia). This review focuses on cardiac channelopathies, which are characterized by lethal arrhythmias in the structurally normal heart, incomplete penetrance, and variable expressivity. Arrhythmias in these diseases result from pathogenic variants in genes encoding cardiac ion channels or associated proteins. Due to a lack of gross structural changes in the heart, channelopathies are often considered as potential causes of death in otherwise unexplained forensic autopsies. The asymptomatic nature of channelopathies is cause for concern in family members who may be carrying genetic risk factors, making the identification of these genetic factors of significant clinical importance.

## 1. Introduction

Sudden death (SD) is defined as an unexpected natural death occurring within an hour after the onset of symptoms. When unwitnessed, SD refers to the death of an individual within 24 h after being seen alive and in a normal state of health [1,2]. Further, the term sudden unexplained death (SUD) is used in cases where an exhaustive postmortem examination fails to determine a conclusive cause of death [3]. Nearly 85% of all SD are of cardiac origin and sudden cardiac death (SCD) is a leading cause of death in Western countries [4,5]. Subjects are otherwise healthy or may have been diagnosed with a disease not severe enough to predict a fatal outcome. In the last ten years, prospective epidemiological studies [6,7,8,9,10] have identified rates between 50 and 100 in every 100,000 deaths [11,12]. Most cases of SCD in patients over 40 years old are the result of coronary heart disease or an ischemic event [13,14]. In contrast, SCD in the young-adult population (<35 years old) is often caused by arrhythmic syndromes with or without structural heart alterations. These diseases result from genetic alternations, which can be inherited [15]. Cardiomyopathies, such as hypertrophic cardiomyopathy (HCM), dilated cardiomyopathy (DCM), and arrhythmogenic cardiomyopathy (AC) are characterized by the presence of structural heart alterations that lead to SCD-related arrhythmias. These arrhythmias are often the result of genetic alterations in proteins found in the sarcomere, desmosomes, or cytoskeleton. Channelopathies, such as long QT syndrome (LQTS), Brugada syndrome (BrS), short QT syndrome (SQTS), and catecholaminergic polymorphic ventricular tachycardia (CPVT) are characterized by malignant arrhythmias in a normal heart resulting from genetic alterations in ion channels or associated proteins. Recent studies have found that about 30% of negative autopsies in young individuals (<15 years) could possibly be explained by pathogenic variations in channelopathy-related genes [16].

In recent years, technological advances in the field of genetics have facilitated the study of a high number of genes in a short period of time. The application of this technology to cardiology has facilitated the identification of several key genes associated with SCD. As a result, genetic testing has been progressively incorporated into clinical diagnosis, assisting with the identification of the cause of disease in clinically affected patients and in unsolved post-mortem cases, as well as identifying risk factors in asymptomatic carriers [17]. These advances have also generated an increasing volume of genetic information that needs to be carefully interpreted, especially in cases where variants of uncertain significance have been identified. In such cases, a multidisciplinary team of clinicians, cardiologists, and genetic counselors works together to appropriately interpret the results both at the genetic and clinical level. Inherited cardiac diseases are characterized by variable expressivity and incomplete penetrance even among family members, representing additional challenges in the interpretation of genetic results. This review describes recent advances in clinical diagnosis and the genetics of cardiac channelopathies.

## 2. Channelopathies: An Overview

The heart is an electromechanical pump electrically triggered by the generation and propagation of an action potential (AP) across myocytes. This is followed by a period of muscle contraction and relaxation until the generation of the next impulse [18,19]. Myocardial AP is generated by ionic changes across the membrane. The sequential activation and inactivation of ion channels that conduct depolarizing, inward currents (Na^+^ and Ca^2+^) and repolarizing, and outward currents (K^+^) enable transmembrane ion currents and, subsequently, AP formation [18,20]. The direction of ion currents is determined by the electrochemical gradient of the corresponding ions. Cardiac ion channel expression and properties are distinct in different regions of the heart, leading to unidirectional propagation of electrical activity. Changes in AP, synchronization, and/or propagation of electrical impulse predispose to potentially malignant arrhythmias [18,19]. These modifications may be induced by pathogenic variants in genes encoding ion channels or associated proteins. As mentioned above, the main cardiac channelopathies associated with SCD are BrS, LQTS, SQTS, and CPVT.

## 3. Brugada Syndrome

Twenty-five years ago, eight individuals sharing the same phenotype were reported to have been resuscitated from SCD caused by documented ventricular fibrillation (VF) showing an ST segment elevation in the right precordial leads in a structurally normal heart [21]. In 1996, the term “Brugada syndrome” was first used to define the “*right bundle branch block, persistent ST segment elevation, and sudden death syndrome*” [22]. Some years later, BrS was reported as “*sudden unexplained nocturnal death syndrome*”, also called *bangungut* in the Philippines, *pokkuri* in Japan, or *lai tai* in Thailand, all characterized by nocturnal death primarily in males around 40 years of age [23]. Currently, the global prevalence of BrS varies from five to 20 in every 10,000 individuals, and there is a strong gender disequilibrium ratio of three to one (male to female) likely due to the influence of hormones [24]. This rate is more than likely underestimated, however, due to the presence of concealed forms of the disease and to ethnic and geographic differences [24]. BrS is endemic in Southeast Asia and is the second cause of death among young men after car accidents [25], showing a male-dominated disequilibrium up to 9:1 [26]. The first manifestation of the disease usually occurs during rest or sleep likely due to high vagal tone [27]. The phenotype is also age-dependent; hence, adults show more abnormalities than adolescents [28] probably also resulting from hormonal differences. In the pediatric population, despite scarce information published to date, the incidence is very low and lacks a male prevalence, perhaps due to the low levels of testosterone found in children of both genders [27].

### 3.1. Clinical Presentation and Diagnosis

BrS is clinically characterized by ST segment elevation in leads V1–V3 of an electrocardiogram (ECG) [29]. Recent reports suggest that BrS could be responsible for 4%–12% of all SD and up to 20% of SD in patients with structurally normal hearts [21]. The diagnosis of BrS is based on clinical and electrocardiographic features and despite continuous advances, incomplete penetrance, and dynamic ECG manifestations confer difficulties in BrS diagnosis. Hence, patients may remain completely asymptomatic or suffer SCD secondary to polymorphic ventricular tachycardia (PVT)/VF [22]. Frequently, SCD can be the first manifestation of the disease [23]. Even though the cause has not been elucidated, structural myocardial abnormalities have been reported in BrS patients [24,25].

**Figure 1:**
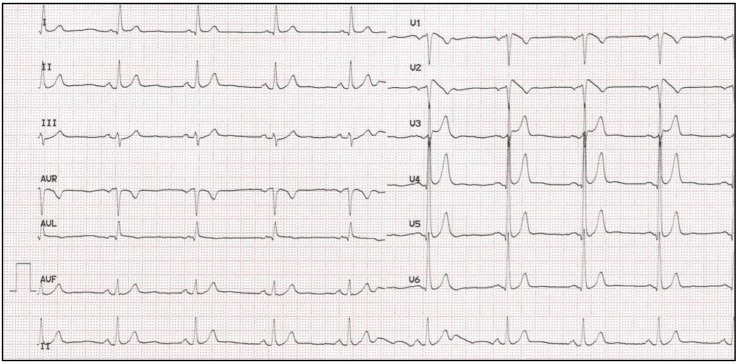
Brugada syndrome type I electrocardiogram (ECG) from a 59-year-old male.

Originally, BrS was reported as “*persistent ST elevation and with right bundle branch block*”, however, both criteria are no longer necessary for diagnosis. Currently, clinical diagnosis requires an ST segment elevation in one of the right precordial leads at baseline or after the use of sodium blockers. Three types of ECG have been described (types I, II and III). Type I is characterized by ST segment elevation followed by a negative T wave, with little or no isoelectric separation, with a “*coved morphology”* [26] (Figure 1). The ECG types II and III present with saddleback-shaped patterns, with a high initial augmentation followed by an ST elevation greater than 2 mm for type II and less than 2 mm for type III. The second Brugada consensus report proposed that only type I is diagnostic for BrS [21] and, in 2013, it was proposed that both a spontaneous type I pattern and a provoked type I pattern (with baseline type II or III pattern) in at least one right precordial lead (V1 or V2) should be considered sufficient for a definitive diagnosis of BrS [27]. This pattern may be spontaneous or induced by a pharmacological test using Class I antiarrhythmic drugs (AAD) [22]. Types II and III ECG patterns are suggestive, but not diagnostic, of BrS. It has also been reported that a type I pattern is observed in nearly 25% of tracings and most ECG will normalize at follow-up. Therefore, the current diagnosis of BrS is based on a type I ECG pattern and any of the following clinical features: documented VF, PVT, inducibility of VT with programmed electrical stimulation, family history of SCD at younger than 45 years of age, covered-type ECG in family members, unexplained syncope, or nocturnal agonal respiration [21].

### 3.2. Genetics

**Figure 2:**
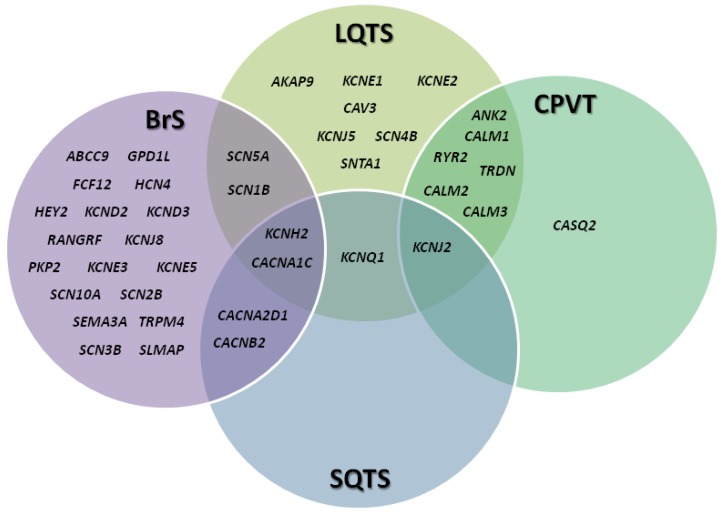
Diagram of the overlap between the genes associated with Brugada syndrome (BrS), short QT syndrome (SQTS), long short QT syndrome (LQTS) and catecholaminergic polymorphic ventricular tachycardia (CPVT).

BrS is a channelopathy with an autosomal-dominant pattern of inheritance. The first genetic alteration associated with BrS was identified in the *SCN5A* gene encoding the α-subunit of the cardiac sodium channel, Nav1.5 [28]. Since then, more than 450 pathogenic variants have been identified in 24 genes encoding sodium, potassium, and calcium channels or associated proteins (*ABCC9*, *CACNA1C*, *CACNA2D1*, *CACNB2*, *FGF12*, *GPD1L*, *HCN4*, *HEY2*, *KCND2*, *KCND3*, *KCNE3*, *KCNE5*, *KCNH2*, *KCNJ8*, *PKP2*, *RANGRF*, *SCN10A*, *SCN1B*, *SCN2B*, *SCN3B*, *SCN5A*, *SEMA3A*, *SLMAP*, and *TRPM4*) [23] (Figure 2).

Approximately 20%–25% of BrS patients are genetically diagnosed with pathogenic variations in *SCN5A*. However, known BrS-susceptibility genes can only explain 30%–35% of clinically diagnosed cases, indicating that 65%–70% of BrS patients remain genetically unsolved [23]. Besides *SCN5A*, pathogenic variations in *SCN1B* [30], *SCN2B* [31], and *SCN3B* [32], which encode the β subunits of the Nav1.5 sodium channel, and *SCN10A* [33], encoding the neuronal sodium channel Nav1.8, have been discovered to modify the sodium channel’s function. While mutations in *SCN1B, SCN2B*, and *SCN3B* are rare, several studies have reported that *SCN10A* mutations account for 2.5%–16% of BrS patients [33,34,35,36].

Calcium channels (*CACNA1C*, *CANB2b* and *CACNA2D1*) have also been reported as BrS-susceptibility genes. Pathogenic variants in *CACNA1C* and *CACNB2*, which encode the α1c and β-2b subunits of the L-type cardiac Ca^2+^ channel, respectively, cause a decrease in I_Ca_ channels and make up nearly 11.5% of BrS cases in which patients present with a syndrome overlapping the typical BrS ECG pattern with a short QT interval [37,38]. *CACNA2D1*, which encodes the α-2/δ subunit of the L-type cardiac Ca^2+^ channel, regulates the current density and activation/inactivation kinetics of the Ca^2+^ channel and is a gene associated with BrS but in a low frequency [39].

Putative gain-of-function mutations in genes encoding channels that conduct outward potassium currents have also been reported in a few BrS cases. Gain-of-function mutations in *KCND3* have been implicated in BrS [40] with an enhanced I_to_ current gradient within the right ventricle where *KCND3* expression is the highest. Gain-of-function mutations in *KCND2* have been associated with J-wave syndromes, including BrS [41]. *KCNE3*, encoding MiRP2, decreases the I_to_ current by interacting with the channel Kv4.3 and results in increased I_to_ magnitude and density in the human heart, which could underlie the pathogenesis of BrS-pattern ECG [42]. *KCNE5* is located on the X chromosome and encodes an auxiliary β subunit for K channels. Mutations in *KCNE5* cause modifications to potassium channels that lead to an increase in the I_to_ current and have been linked to BrS [43]. Functional KATP channels have an octameric subunit structure with four pore-forming subunits (Kir6.1) encoded by *KCNJ8* and four sulfonylurea receptors (SUR2A) encoded by *ABCC9*. Pathogenic variants in *KCNJ8* or ABCC9 may result in a severe arrhythmic phenotype typical of BrS [44]. Mutations in *KCNH2* have mainly been associated with LQTS; however, a few pathogenic variants have also been reported in patients with a short-QT interval and Brugada ECG [45,46].

Genes encoding proteins that interact with sodium, calcium, and potassium channels have also been reported as being associated with BrS. *RANGRF,* which encodes MOG1, can impair the trafficking of Nav1.5 to the membrane, leading to I_Na_ reduction and clinical manifestation of BrS [47]. *GPD1L* may affect trafficking of the cardiac sodium channel to the cell surface and regulate cardiac sodium current [48], but has only been implied in a small number of BrS cases [49]. *SLMAP*, which is found in T-tubules and in the sarcoplasmic reticulum and has an unknown function, is known to cause BrS by modulating the intracellular trafficking of the Nav1.5 channel [50]. Pathogenic variations in *PKP2*, the primary gene responsible for arrhythmogenic right ventricular cardiomyopathy, have also been recently associated with BrS [51,52]. Pathogenic mutations in *TRPM4* alter the resting potential of the membrane and changes in the function of this channel may reduce the availability of sodium channels, ultimately leading to BrS [53]. *FGF12* is the major fibroblast growth factor homologous factor expressed in the human ventricle and is implicated in an Na^+^ channel loss-of-function phenotype consistent with BrS diagnosis [54]. *HEY*2, which encodes the transcriptional repressor hairy/enhancer-of-split related to YRPW motif protein, has been found to play a role in the regulation of *SCN5A* expression and conduction velocity in the heart, suggesting that BrS may originate from altered transcriptional programming during cardiac development [55]. *HCN4* encodes the hyperpolarization activated cyclic nucleotide-gated potassium channel 4, a voltage-gated ion channel mediating the pacemaker current in the heart, and although its causative role remains unclear, it has been reported in a few patients with BrS [56]. The *SEMA3A* gene is a protein that inhibits Kv4.3 and is associated with the BrS gene through a Kv4.3 gain-of-function mechanism [57]. Concerning copy number variation (CNV), one large-scale deletion of the *SCN5A* gene was identified as a cause of the disease [58] and a recent study identified a duplication in one of 220 patients analyzed (0.45%) [59]. However, several reports conclude that genetic imbalances are uncommon in BrS families [60,61,62]. Despite the recent improvements in BrS diagnosing, only 30%–35% of cases are genetically diagnosed; 25%–30% carry a pathogenic variant in *SCN5A* [63].

It is important to notice that, except for *SCN5A* and *GPD1L*, most of the genes associated with BrS susceptibility have been identified only in single patients, in a few unrelated patients, or in small families through candidate gene analysis. Therefore, further investigation is needed before genes are implicated in the pathogenesis of BrS or any other disease and in order to avoid false-positive reports of causality in the context of genetic counseling [64]. Consequently, current clinical guidelines only recommend genetic analysis of the *SCN5A* gene [65].

## 4. Long QT Syndrome

More than 50 years ago, a family with concomitant deafness, mutism, and a peculiar heart disease was described [66]. The ECG revealed a pronounced prolongation of the QT interval in all cases. Three of the deaf-mute children died suddenly at ages four, five, and nine. One year after, in 1958, Levine et al. reported a case of an 8-year-old boy who died suddenly after having been previously diagnosed with congenital deaf-mutism, attacks of unconsciousness, and an ECG with a prolonged QT interval and large T waves, but no other objective evidence of organic heart disease or any other diseases were observed upon post-mortem examination [67]. Shortly thereafter, Romano et al. [68] and Ward [69] published QT prolongation in one parent and several children from two different families, all of whom possessed normal hearing but experienced recurrent syncope and SD. Since 1975, the unifying name of “long QT syndrome” has included both the Jervell-Lange-Nielsen and the Romano-Ward syndromes, associated with and without deafness, respectively [70].

LQTS can be congenital or acquired. While congenital LQTS is associated with mutations in ion channels and/or associated proteins [71], acquired LQTS is generally associated with drugs and electrolyte imbalance (hypokalemia, hypocalcaemia, and hypomagnesaemia). The prevalence was assumed to be between 1/5000 and 1/20,000, but a study performed in children suggested that the prevalence of LQTS in infants is closer to 1/2000 [72]. Gender is a major factor in determining the course and clinical manifestation of the LQTS. Even though the QT interval duration is similar between young boys and girls, differences appear during puberty in which boys present with a shorter QT interval [73]. In congenital LQTS, women have longer QT intervals than men [74,75]. Therefore, women are more often clinically diagnosed than men despite equal genotype sex-distribution [76]. Curiously, the probability of a first cardiac event is higher in males by age 15 but decreases after puberty [74,77]. Sex hormones are suggested to play an important role in conferring these gender differences; different phases of the menstrual cycle, pregnancy, and the postpartum period are all associated with changes in QT duration and the incidence of PVT in LQTS patients [76,78]. However, the first cardiac event tends to be more often fatal in males than in females [77]. Additionally, in response to QT prolonging drugs, women are also more at risk of developing arrhythmias than men [79,80,81].

### 4.1. Clinical Presentation and Diagnosis

**Figure 3:**
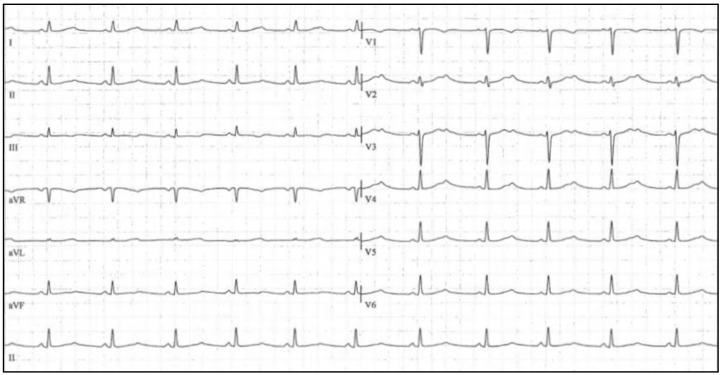
ECG from a 20-year-old patient with LQTS.

LQTS is an inherited arrhythmia characterized by a prolonged QTc interval in the 12-lead ECGs (with QTc values >470 ms for males and >480 ms for females, representing approximate 99th percentile values) (Figure 3). The clinical manifestations of LQTS can be variable, ranging from asymptomatic patients diagnosed through family screening, to SCD, syncope, convulsions, malignant ventricular arrhythmias, VF, and typically *torsade de pointes* [82,83,84,85,86,87]. SD usually occurs in healthy children and teenagers and in physically or emotionally stressful situations. LQTS is a cardiac channelopathy characterized by prolonged ventricular repolarization and life-threatening arrhythmias and displays incomplete penetrance and variable expressivity [88]. All symptomatic individuals should be treated, as there is a high lethality among symptomatic and untreated patients [27]. Exceptions exist and patients with modest or normal QT intervals can also experience symptoms. However, in general, the longer QT interval increases the risk of malignant arrhythmias. In addition, there is evidence that risk of malignant arrhythmia increases when QTc exceeds 500–550 ms [89,90].

The diagnosis of LQTS is based on clinical and electrocardiographic features. In 1985, Schwartz et al. published the first description of diagnostic criteria for LQTS, which remain essentially valid for quick assessment [91]. However, the approach became quantitative with the presentation in 1993 of a diagnostic score known as the “Schwartz score” [92], which has since been updated [93,94]. Points are assigned based on ECG, clinical history, and family history criteria. As such, the diagnosis of LQTS is established by a “Schwartz score” of ≥3.5 without a secondary cause for QT prolongation and/or by the presence of a QTc interval ≥500 ms in repeated ECGs without a secondary cause for QT prolongation and/or by the presence of a pathogenic variant in one of the genes known to be associated with LQTS [27]. Additionally, LQTS can be diagnosed if QTc is between 480 and 499 ms in patients with unexplained syncope without a secondary cause for QT prolongation and in the absence of a pathogenic genetic variant [27].

A careful analysis of T-wave morphology can also provide useful diagnostic information as each of the major LQTS genotypes correlates with specific ST-T wave patterns [95,96]. For example, LQT1 has a broad-based T wave; a low-amplitude and notched T wave are characteristic of LQT2; LQT3 has a late-appearing T wave [95]; and LQT7 has a mild QT prolongation with a prominent U wave [97]. Moreover, certain genotypes may be associated with changes in heart rate [98] and prominent U waves and T-U complexes are commonly identified [85]. Furthermore, T-wave alternation is a sign of electrical instability and can be a precursor to ventricular tachycardia (VT) or fibrillation [99,100,101].

### 4.2. Genetics

The genetic heterogeneity of LQTS has led to its classification into subtypes based on genetic loci. In 1991, a linkage analysis was performed in a multigenerational family with many affected relatives and the genetic defect was mapped to the small arm of chromosome 11 [102]. However, in 1994, linkage analysis in other cohorts identified loci on chromosomes 7 and 3, demonstrating that not all related individuals with LQTS share the same locus [103,104,105]. To date, pathogenic variants associated with LQTS have been identified in 19 genes: 15 following an autosomal-dominant pattern of inheritance (*AKAP9*, *ANK2*, *CACNA1C*, *CALM1*, *CALM2*, *CALM3*, *CAV3*, *KNCE2*, *KCNH2*, *KCNJ2*, *KCNJ5*, *RYR2*, *SCN1B*, *SCN4B*, *SCN5A* and *SNTA1*), one following an autosomal-recessive pattern (*TRDN*), and two following both autosomal-dominant and -recessive patterns (*KCNQ1* and *KCNE1*) (Figure 2).

Approximately 85% of clinically diagnosed patients have a mutation in one of these genes [106]. The three major LQTS-susceptibility genes are *KCNQ1*, *KCNH2*, and *SCN5A*, and mutations in these genes are associated with about 75% of patients with a clinical LQTS diagnosis. The remaining 16 genes are responsible for nearly 10% of LQTS cases. *KCNQ1* (LQT1) encodes the α-subunit of the voltage-gated potassium channel and mediates the slow component of the delayed rectifier potassium current (*I*_Ks_) [107]. Pathogenic variations in *KCNQ1* reduce *I*_Ks_, prolonging the repolarization phase of the AP [108,109]. The *KCNH2* gene encodes the α-subunit of the voltage-gated potassium channel and mediates the rapidly activating component of the delayed rectifying potassium current (*I*_Kr_). Pathogenic variations in *KCNH2* result in reduced *I*_Kr_ and delayed cardiac repolarization leading to a prolonged QT interval [110]. Gain-of function variants in *SCN5A* (LQT3) induce an increased late inward Nav1.5 current that slows cardiac repolarization, also causing a prolonged QT interval [111]. All other known LQTS-associated genes are responsible in about 10% of LQTS cases. The *ANK2* gene (LQT4) encodes the protein ankyrin-B which is involved in the coordinated assembly of the Na/K ATPase, Na/Ca exchanger, and the inositol triphosphate receptor. A decrease in the role of ankyrin-B alters calcium homeostasis, prolonging repolarization [112]. The first auxiliary proteins implicated in the pathogenesis of LQTS through their modulatory effect on *I*_Ks_ and *I*_Kr_ were *KCNE1* (LQT5) and *KCNE2* (LQT6), respectively [113,114]. *KNCE1* encodes the β-subunit of Mink and *KCNE2* the β-subunit of MiRP1. LQT7, or Andersen–Tawil syndrome, is caused by loss-of-function mutations in *KCNJ2*, which encode for the inward rectifier potassium channel (*I*_K1_). Andersen–Tawil syndrome is a form of LQTS accompanied by extracardiac manifestations that include dysmorphic physical features and periodic paralysis [115,116].

LQT8 occurs due to gain-of-function variants in the *CACNA1C* gene that induce slowed inactivation of Cav1.2. This fact induces an increased influx of calcium, prolongation of AP, and arrhythmias [117,118]. Several point mutations have been described in *CACNA1C* as being associated with a rare multi-systemic syndrome called Timothy syndrome [119,120,121]. Timothy syndrome is characterized by several physical and/or developmental abnormalities in addition to the classic phenotype of QT prolongation and an increased risk of SCD. *CAV3* (LQT9) encodes the Caveolin-3 protein that may play a role in the compartmentalization and regulation of resident ion channels in the caveolae. Abnormalities in *CAV3* have the potential to modify Nav1.5 in a similar manner to that observed with LQT3 through an increased late inward current [122]. This also occurs with pathogenic variants in *SCN4B* (LQT10), which encodes a β-subunit of the sodium channel [123], and pathogenic variants in *SNTA1* (LQT12), which encodes the alpha1-syntrophin protein [124]. *AKAP9* encodes the kinase-A anchor protein-9 and mutations in this gene (LQT11) impair *I*_Ks_ increase, leading to a clinical phenotype similar to that of LQT1 and LQT5 [125,126]. Pathogenic variants in *KCNJ5*, which encodes the inwardly-rectifying potassium channel, result in reduced membrane expression of the protein [127].

Mutations in *CALM1* (LQT14), *CALM2* (LQT15), and *CALM3* (LQTS16), encoding calmodulin, disrupt calcium-ion binding to the protein [128,129,130]. A recent study identified a mutation in *SCN1B* in a LQTS patient and demonstrated that the *SCN1B* mutation increases late sodium current [131]. A long QT interval has been also associated with a patient carrying a mutation in the cardiac ryanodine receptor gene *RYR2* [132]. *TRDN*, which encodes triadin protein, has been identified as a novel autosomal-recessive LQTS-susceptibility gene. Additionally, pathogenic variants in *KCNQ1* (JLN1) and *KCNE1* (JLN2) have also been identified as autosomal-recessive forms of Jervell and Lange–Nielsen syndrome attributable to a decrease in the *I*_Ks_. Jervell and Lange-Nielsen syndrome is characterized by neurosensorial deafness and a markedly prolonged QT interval [133].

## 5. Short QT Syndrome

In 2000, Gussak et al. reported two probands with idiopathic familial persistently short QT intervals [134]. Three years later, Gaita et al. reported six patients from two unrelated families with very short QT intervals at ECG, syncope, palpitations, and a strong family history of SCD [135]. SQTS is a rare channelopathy with an estimated prevalence of less than 1 in 10,000 [136,137,138,139]. Currently, SQTS has been described in only a few families worldwide and all probands present with a QTc below 320 ms without evident structural heart disease [140]. The largest available case series published so far showed that most patients have experienced symptoms and that the manifestation of an abbreviated repolarization was predominant in males [136]. However, even if QTc intervals are significantly longer in females than in males, women should not be regarded as low-risk patients because the risk of experiencing cardiac arrest appears to be similar in males and females. Additionally, SQTS shows a peak of incidence during the first year of life, followed by a quiescent phase encompassing adolescence and another peak at old age [136,141].

### 5.1. Clinical Presentation and Diagnosis

SQTS is a rare inheritable cardiac channelopathy characterized by abnormally short QT intervals and an increased propensity to develop atrial and ventricular tachyarrhythmia in the absence of structural heart disease. Cardiac arrest seems to be the most frequent symptom (up to 40%) [136]. Palpitations are a common symptom (30%), followed by syncope (25%) and atrial fibrillation (AF), which are the first symptoms of the disease in up to 20% of patients. The episodes may occur in a wide range of situations such as in reaction to loud noise, at rest, during exercise, and during daily activity [142]. Some patients show additional QT shortening during bradycardia. To date, there is no evidence to assume that a shorter QTc interval could predispose to a higher risk of ventricular arrhythmias. In fact, the prognosis of patients with asymptomatic SQTS remains undefined. SQTS can be congenital or acquired, with the latter associated with hypercalcemia, hyperkalemia, acidosis [143], and drugs [144].

**Figure 4:**
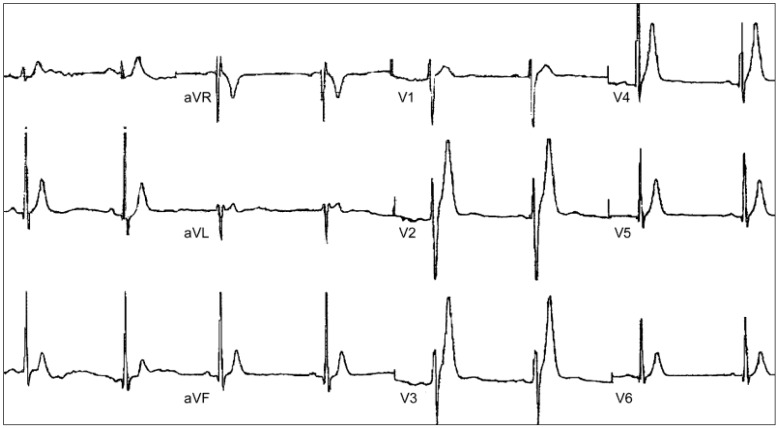
ECG from a patient with SQTS.

ECG constitutes the basis of diagnosis (Figure 4). However, SQTS diagnosis should be based on several findings, including a short QTc interval (≤360 ms in males; ≤370 ms in females) [145,146,147], syncope, episodes of VF or PVT, family history of short QT interval, syncope or VF, occurrence of AF, and no obvious heart disease or extracardiac conditions that abbreviate QT interval [85]. Additionally, there are several other ECG findings that may facilitate the correct diagnosis of SQTS such as the presence of tall, peaked, symmetrical, and narrow-based T waves, prominent U waves [148], depression of the PQ segment [149], or a QRS complex directly followed by a T wave [150].

### 5.2. Genetics

SQTS is a channelopathy with an autosomal-dominant pattern of inheritance and high penetrance. Currently, genetic alterations associated with SQTS have been identified in six genes (*KCNQ1*, *KCNH2*, *KCNJ2*, *CACNA1C*, *CACNB2* and *CACNA2D1*) (Figure 2). Despite the fact that a familial association is present in the majority of patients, the yield of genetic screening is low and varies between 15% and 40% [140,151,152]. The most prevalent subtype of SQTS is associated with gain-of-function mutations in *KCNH2* (SQTS1) that increase current flow through the channel and shorten the AP duration and QT interval [135,153]. Pathogenic variants in *KCNQ1* (SQTS2) increase the repolarizing current, shortening the QT interval [108]. SQTS3 occurs secondary to gain-of-function mutations in *KCNJ2*, leading to an increase in the outward *I_K1_* current and an acceleration of the final phase of repolarization [154]. The QT interval can also be shorted by a reduction in depolarizing currents. Loss-of-function mutations in the α1-, β2-, and α-2/δ subunits of the L-type calcium channel (*CACNA1C* -SQTS4-, *CACNB2* -SQTS5-, and *CACNA2D1* -SQTS6-, respectively) are associated with a shortening of the QT interval and precordial ST elevation reminiscent of BrS [37,155].

## 6. Catecholaminergic Polymorphic Ventricular Tachycardia

CPVT is a pathological condition whereby intense physical exercise or acute emotional stress can trigger abnormal heartbeat—i.e., ventricular tachycardia—that can lead to dizziness, fainting (syncope), and in worst cases to cardiac arrest and sudden death. In 1960, Berg [156] described three sisters with multifocal ventricular extrasystoles without any other signs of structural heart disease. In two of the children, the arrhythmia was accompanied by Adams–Stokes syndrome and one died suddenly. The direct cause of these attacks was believed to be VT or VF. In 1975, the electrophysiological and hemodynamic findings in a six-year-old girl with bidirectional tachycardia were not caused by digitalis, but precipitated by effort and emotion [157]. Following the description of “catecholaminergic polymorphic ventricular tachycardia”, the first comprehensive study of CPVT was published by Leenhard et al. in 1995 [158]. They described 21 children suffering from stress- or emotion-induced syncope, with no evidence of structural heart disease and normal QT intervals. Seven patients presented with a family history of syncope or SD, suggesting a genetic origin of CPVT.

CPVT is a rare disease with an estimated prevalence of 1:10,000 [159,160]. CPVT commonly manifests at an early age and has poor spontaneous outcome [161]. Gender plays an important role in the etiology, pathogenesis, and cardiac risk stratification of patients with CPVT. Earlier onset of clinical symptoms and a significantly higher risk of cardiac events at a young age is observed in males [162].

### 6.1. Clinical Presentation and Diagnosis

CPVT is characterized by polymorphic premature ventricular contractions or polymorphic ventricular tachyarrhythmias in genetically predisposed individuals under physical or emotional stress. Syncope is the first clinical manifestation of CPVT patients and less prevalent signs and symptoms include dizziness or palpitations [163]. Presentation of most arrhythmic events occurs during childhood, between seven and 11 years, and more than 60% of affected individuals have experienced a syncopal episode or cardiac arrest by age 20 [164]. Generally, there is a two-year delay between the first and second syncope episode in patients with CPVT. Family history of juvenile SCD and/or stress-related syncope is present in approximately 30% of patients [165]. CPVT is one of the most malignant and yet insufficiently recognized primary electrical diseases of the heart. It exhibits incomplete penetrance, which has been reported to be around 78% [166], and has variable expressivity.

**Figure 5:**
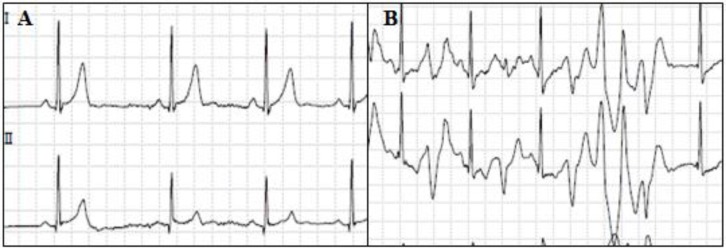
Exercise ECGs from a patient with CPVT. (**a**) Basal; (**b**) Bidireccional ventricular arrhythmia.

The basal ECG of patients with CPVT tends to be normal, although some authors have reported lower-than-normal heart rates, and others have observed prominent U waves [167]. Diagnostic characteristics of CPVT are unmasked by exercise ECG [168] (Figure 5). Usually, the beginning of ventricular arrhythmias is 100–120 beats/min [169]. In situations where exercise persists, the premature ventricular complexes may progress to bigeminy and non-sustained ventricular tachyarrhythmia; if exercise is maintained, the duration of VT progressively increases and may become sustained [170].

### 6.2. Genetics

CPVT is a channelopathy with both autosomal-dominant and, less frequently, autosomal-recessive inheritance patterns. The first CPVT-associated variants were identified in 2001 in the gene encoding the cardiac ryanodine receptor (*RYR2*) in four of 12 probands presenting with typical CPVT in the absence of structural heart abnormalities [171]. Since then, approximately 150 different *RYR2* mutations have been associated with CPVT and pathogenic variants in *RYR2* account for approximately 60% of individuals with clinical diagnosis of CPVT. Other less prevalent genes associated with CPVT are *CASQ2*, *CALM2*, *CALM3*, *TRDN*, and possibly *ANK2*, *KCNJ2* and *CALM1* (Figure 2). Altogether, these genes are responsible for an additional 5% of cases. Anomalies in *CASQ2*, which encodes the calsequestrin 2 protein, cause the second most common type of CPVT as a result of increased calcium release from the sarcoplasmic reticulum. Pathogenic variants in *CASQ2* are associated with an autosomal-dominant pattern of inheritance, causing a higher rate of SD than that observed with *RYR2* mutations [172]. However, *CASQ2* variants have also been reported to be associated with autosomal-recessive CPVT [173].

Calmodulin is a protein encoded by *CALM1*, *CALM2* and *CALM3* that is involved in the calcium-dependent ICa inactivation of the L-type Ca channel and stabilizes the ryanodine channel. Therefore, pathogenic variants in calmodulin may cause Ca^2+^ overload [174]. Pathogenic variants in *CALM1* may demonstrate compromised calcium binding and an aberrant interaction with the *RYR2* calmodulin-binding-domain peptide [175]. In addition, pathogenic variants in *CALM2* also cause reduced Ca^2+^-binding affinity and can be associated with overlapping features of LQTS and CPVT. *CALM3* has now been associated with CPVT and both *CALM1* and *CALM3* mutations evoke arrhythmogenic Ca disturbances via ryanodine receptor 2 dysregulation [176]. Candidate gene screening has implicated *TRDN* as an autosomal-recessive form of CPVT [177]. *TRDN* encodes the triadin protein, which connects calsequestrin to ryanodine receptor 2 and stabilizes the channel. Pathogenic variants in *TRDN* may result in a diastolic leak of Ca^2+^ and Ca^2+^ overload in the myocytes. Both *KCNJ2* [178] and *ANK2* [179] pathogenic variants are also reported in patients with exercise-induced bi-directional VT. Finally, a locus for a severe form of CPVT was mapped at chromosome 7p22-p14 (homozygous) in a family but the responsible gene has not been identified [180].

## 7. Conclusions

SCD remains a major cause of death, mainly in young populations. In the last few years, technological improvements in genetics have helped in both the diagnosis and prevention of SCD. To date, several genes have been implicated in ion channel diseases, but a large number of families remain without a recognized genetic cause. The primary challenges to SCD prevention are early identification of individuals at risk and clinical measures in asymptomatic individuals carrying a mutation, since the first manifestation of the disease can be SCD itself. In the future, comprehensive genotype-phenotype studies in large cohorts of families should be performed in order to clarify the genetic basis of SCD-related diseases as well as the adoption of personalized preventive therapies for the prevention of SCD. Close interaction between families and a team of specialists including cardiologists, geneticists, genetic counselors, and even psychologists will be crucial to the development of such therapies.
